# Design and Computational Modeling of Fabric Soft Pneumatic Actuators for Wearable Assistive Devices

**DOI:** 10.1038/s41598-020-65003-2

**Published:** 2020-06-15

**Authors:** Pham Huy Nguyen, Wenlong Zhang

**Affiliations:** The Polytechnic School, Ira A. Fulton Schools of Engineering, Arizona State University, Mesa, AZ 85212 USA

**Keywords:** Engineering, Soft materials

## Abstract

Assistive wearable soft robotic systems have recently made a surge in the field of biomedical robotics, as soft materials allow safe and transparent interactions between the users and devices. A recent interest in the field of soft pneumatic actuators (SPAs) has been the introduction of a new class of actuators called fabric soft pneumatic actuators (FSPAs). These actuators exploit the unique capabilities of different woven and knit textiles, including zero initial stiffness, full collapsibility, high power-to-weight ratio, puncture resistant, and high stretchability. By using 2D manufacturing methods we are able to create actuators that can extend, contract, twist, bend, and perform a combination of these motions in 3D space. This paper presents a comprehensive simulation and design tool for various types of FSPAs using finite element method (FEM) models. The FEM models are developed and experimentally validated, in order to capture the complex non-linear behavior of individual actuators optimized for free displacement and blocked force, applicable for wearable assistive tasks.

## Introduction

In the recent years, soft robotics has emerged as a candidate to create novel robotic systems with pre-programmable capabilities, while capable of withstanding large deformations. These systems have shown to be potentially useful in diverse application fields ranging from bio-inspired robotic systems^1,2^, adaptable locomotion in unstructured environments^1,3^, grasping/manipulation of objects^4^, invasive surgical instruments^5^ and assistive/rehabilitative devices^6^.

These intrinsically soft robots have advantages over conventional rigid robots by being low-cost, lightweight, highly compliant, and inherently safe when interacting with the unknown environment and human body^2,6^. Therefore, these soft robots can be utilized for rehabilitation, prevention of injuries, or augmentation of the capabilities of healty individuals^6,7^.

Soft wearable assistive/rehabilitative robots are generally categorized based on the joints they assist as well as the type of actuators actuators utilized to design them^6^. Upper-body soft wearable robots have been developed to actively support fingers^8–13^, wrists^14^, elbows^15,16^, shoulders^17–19^, necks^20^, forearms^21,22^, and spines^23,24^. Lower-body soft wearable robots have provided assistance to the hips^25^, knees^26,27^, and ankles^28–31^. Common soft actuation methods for assistive/rehabilitative tasks include cable-driven^11,14,25^, origami^32,33^, and soft pneumatic actuators (SPAs)^2,6,34^.

SPAs broadly categorizes soft actuators that require positive or negative pressure to generate pre-programmable motion^2,6,34^. Pneumatic artificial muscles^20,29^, elastomeric^10,23,35^ and inflatable fabric soft pneumatic actuators (FSPAs) all fall under this category^9,13,18,19,21,22,26–28,36,37^. SPAs can also be further classified according to how they are mechanically programmed to move whether in the macro or micro-scale^2,6,34^. Their motion paths can be programmed using combinations of multiple inflatable chambers or actuators as seen with peano muscles and bellow actuators^15,37–50^. A form of external/internal flexible mechanical metamaterials^51^ (for example, reinforcements^6,34,52–54^ and auxetic structures^55–58^) or origami structures^32,33,59–62^ can also be used for mechanical programmable motions. The programmable motions include^2,6,34,42,54,63^: twisting^64,65^, bending^39,50^, stiffening^26,28^, contracting^30,39,41,44,46,66^, or extending/growing^67–70^ in space. Further, by combining multiple actuators together in a modular unit, a continuum, multi-chambered and multi-DOF actuator can be created^38,50,71,72^.

The development of wearable technologies has generated a lot of interest in the use of textiles or fabrics, both terms used interchangeably in this work, due to their versatility, repeatable production, and omnipresent nature^73^. Fabrics have also shown to be a promising medium to incorporate functionalities like: soft computing, flexible electronics, energy harvesting, sensing and actuation^73^. Soft fabric actuation has shown possibilities of utilizing fabric to generate movement and provide assistance^74^. The construction of these fabric actuators has been through either intrinsic or extrinsic modifications of the materials^74^.

Wearable assistive devices have seen a growth in utilizing extrinsically-modified fabric actuation technology^9,13,16,28,36,37^. Extrinsically-modified fabric actuators, are fabricated by superficially attaching active materials on the surface of the substrate fabric, for example laminating thermoplastic polyurethane (TPU) material on the substrate fabric to create FSPAs^73,74^. This paradigm shift has lead to design of SPAs that are easily integrated with or hidden underneath the users’ clothes. Along with the ease of fabrication, wearability, pliability, and availability, these actuators also provide enough torque and force assistance to the extremity, making this technology more adoptable for everyday life^8,9,12,13,15–18,21,22,26,28,31,37,75^.

**Figure 1:**
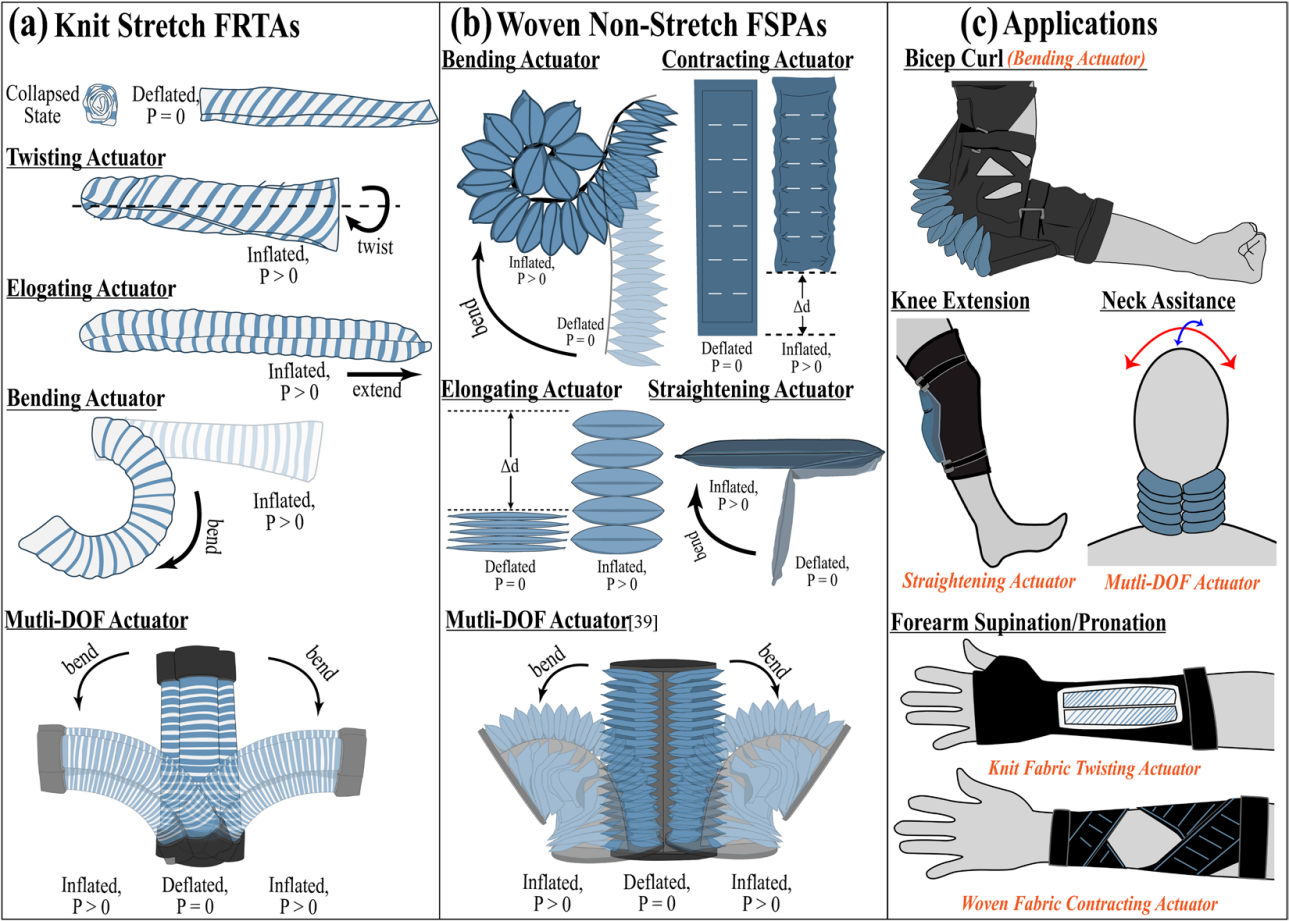
(**a**) Illustrated concept of the knit stretch FSPAs. (**b**) Illustrated concept of the woven non-stretch FSPAs. (**c**) Illustrated concept of the various wearable assistive applications using FSPAs.

FSPAs are further classified based on the types of fabrics used to make them. In this work, we focus on two categories of extrinsically modified FSPAs, woven and knit FSPAs, shown in Fig. 1a,b. Because of how each type of fabric material is manufactured, woven fabrics are generally puncture resistant but less deformable, while knit fabrics are easily deformable and have an innate mechanical anisotropy (showing variable stretchability in bi-directions)^73^. Recent research have seen woven fabrics used to create highly robust twisting, contracting and bending actuators^12,13,15,18,19,24,27–31,37,43,50,75^, as well as the use of the knit textiles to create bending actuators for grippers and wearable robots^8,9,76^.

There have been various computational and analytical studies on the prediction of fabric properties at the fiber or yarn level, but not for the entire set of the fabric structural hierarchy^73^. Only recently have models for woven FSPAs been developed to predict their force and motion capabilities^8,22,28^. Our preliminary work has shown promise in utilizing computational models for woven FSPAs for the elbow^15^ and also continuum assistive robots^50^. On the other side of the spectrum, modeling of knit FSPAs are still in the nascent phase of development^8,9^.

In this paper, we further investigate the combination of various textile layers to mechanically program actuators in order to perform various motion profiles, as highlighted in previous work^15,24,26,36,50,75^. Specifically, two categories of multi-material and multi-layered woven and knit FSPAs as shown in Fig. 1, are studied and fabricated. A comprehensive material study of both the various woven and knit anisotropic textiles are conducted for large deformations to generate material models. To accurately predict the complex mechanical response of the FSPAs, we opt to create computational finite element method (FEM) models. Computational FEM models have the ability to generate detailed models, based on the actuator’s variable geometrical parameters non-linear behaviors and capture the detailed stress-strain distributions of multi-material and multi-layered^7,23,77^. We develop an all-inclusive design tool using the computational models, that will benchmark the design criteria for developing a new robust woven or knit fabric actuator based on the desired geometrical parameters and application force/torque requirements. This comprehensive tool will allow for scalability and customizability of diverse FSPAs prior to fabrication.

## Design and Fabrication of the FSPAs

**Figure 2:**
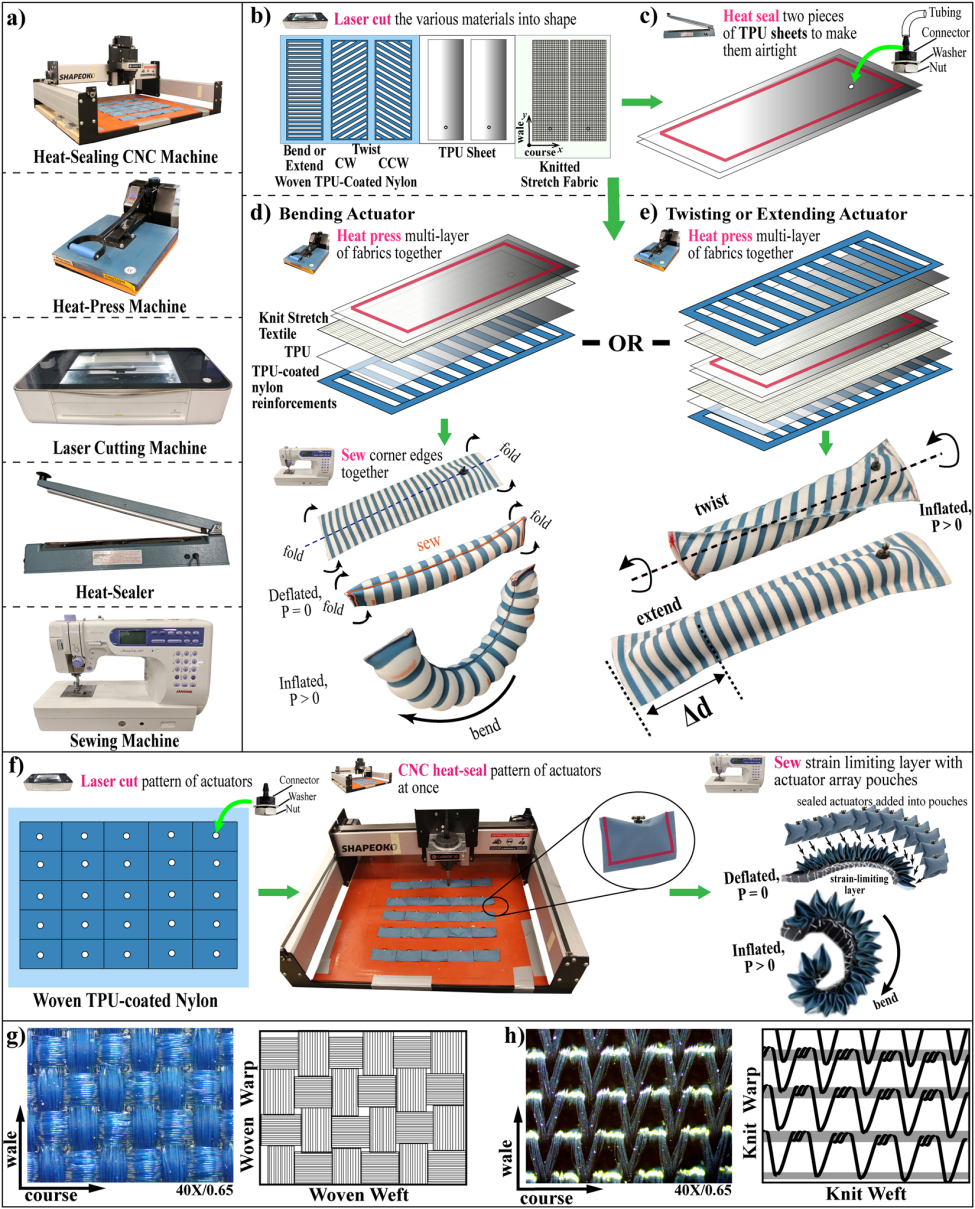
(**a**) Machines used for fabrication (**b**) Layout of fabrics cut using a laser cutter (**c**) Fabrication procedure of internal TPU bladder (**d**) Fabrication procedure of knit stretch FRTA for bending. (**e**) Fabrication procedure of knit Stretch FRTA for twisting and extending. (**f**) Fabrication procedure for woven fabric actuators. (**g**) Microscope view and illustration of the yarns in the woven fabric. (**h**) Microscope view and illustration of the yarns in the knit stretch fabric.

The two main fabrics used in this work include, the woven non-stretch thermoplastic polyurethane (TPU)-coated nylon fabric (6607, Rockywoods Fabric, Loveland, CO) and the bi-directional high-stretch knitted fabric (24350, Darlington Fabrics, Westerly, RI). Both fabrics are seen under a microscope (OMAX A355U, OMAX Microscope, Seattle, WA) with a magnification factor of 40× and numerical aperture of 0.65, as shown in Fig. 2g, h. The two directions of stretch include the wale (in the $y$-direction) and the course (in the $x$-direction).

Woven fabrics are generally created with vertical (warp) yarns interlaced with horizontal (weft) yarns in a checkered pattern, as seen in Fig. 2g^73^. Material properties of woven fabrics are dependent on the strain properties of the yarns used to create them. The nature of the weaving method, creates a tight interconnected thread system resulting in a more stable, rigid, and difficult to deform fabric^73^. On the other hand, knit fabrics are created by the interlocking loops of a single yarn (i.e. weft knits) or multiple yarns (i.e. warp knits)^73^. The knitted fabric used in this work, is created by using a warp knitting. The fabric is made of 83% semi-dull nylon and 17% spandex. This essentially means that warp knits often have mechanical anisotropy, because one stretch direction is relatively stretchier than the other (the preferential strain direction), as seen in Fig. 2h. Thus, the knit fabrics show high bi-directional stretchability and elastic recovery, comparable to the hyperelastic properties of elastomers.

These woven and knit fabrics are used to create two categories of FSPAs: woven FSPAs highlighted in Fig. 1a, and knit fabric-reinforced textile actuators (FRTAs) as highlighted in Fig. 1b. The woven non-stretch fabric actuators generate motion by combining multiple pouch fabric actuators, that inflate to a set size, in various array formations, to contract, straighten, bend, or elongate. In contrast, the knitted FRTAs are developed by combining an internal knit fabric shell with strain-limiting woven fabric reinforcement layers, so the fabric’s overall anisotropic behavior can be augmented during pressurization. Further, the woven fabric reinforcements also reduce the local stresses and strains on the internal shell and minimizes any surface damage, from abrasion commonly seen with the use of Kevlar threads as reinforcements, seen in previous work^6^. Finally, by arranging multiple actuators in different orientations we can also create multiple degree-of-freedom (DOF) actuators, as shown in Fig. 1. These various types of actuators generate motion profiles that can serve various target applications in the field of wearable assistive devices as featured in Fig. 1c and further mentioned in Supplementary Table 1.

### Fabrication of the FSPAs

The machines used in the fabrication procedure are shown in Fig. 2a. The laser-cutter (Glowforge Prof, Glowforge, Seattle, WA) is used to cut all the TPU (Fastelfilm 20093, Fastel Adhesive, Clemente, CA), woven and knit fabrics into the desired geometry, as shown in Fig. 2b. The TPU sheets are used to bond the knit fabric and the woven fabric reinforcements, while coating the knit fabric substrate to make it airtight. However, air leakage through the skin of the fabric is still noticed. Therefore, an additional airtight TPU bladder with a pneumatic connector (5463K361, McMaster-Carr, Elmhurst, IL), is still made using an impulse sealer (751143, Metronic, Seattle, WA) as seen in Fig. 2c.

There are two variations of fabricating the FRTAs, one for FRTAs that perform bending, in Fig. 2d, and the other FRTAs that elongate and/or twist, as shown in Fig. 2e. In the first variation of the fabrication method the knit stretch fabric, a single TPU sheet, and woven TPU-coated reinforcements are assembled and bonded all at once using a heat press (FLHP 3802, FancierStudio, Hayward, CA). The TPU bladder is placed in the middle of the prepared multi-layered fabric set, and the structure is folded and sewn, using a super-imposed seam along the center. The sewn portion creates the strain-limiting, inextensible seam to encourage bending towards that particular direction. In the second variation of the fabrication method, two sets of knit stretch fabric and woven reinforcements are created. The additional TPU bladder is placed between the two sets of multi-layered fabric sets and the edges of the layers are heat-sealed or sewn along the edges using high-stretch elastic thread (Maxi Lock Stretch, American & Efird, Mount Holly, NC). Different clockwise/counterclockwise twisting and elongating actuators can be developed by varying the angle of woven reinforcements.

In order to fabricate the woven FSPAs, the TPU-coated nylon fabric is cut into the desired geometries as seen in Fig. 2f. The woven TPU-coated nylon already has a side pre-laminated with a TPU coating to allow bonding. Pneumatic fittings are attached to the cutouts and aligned on the bed of the customized computer numerical control (CNC) router (Shapeoko 3, Carbide Motion, Torrance, CA) with a soldering iron tip set at 230 °C. The CNC router traces and seals the fabric cutouts to seal the individual fabric actuators. This procedure can instantly create the woven straightening or contracting FSPAs. In order to create the woven bending and elongating FSPAs, pouches with the same size as the actuators are created. The pouches are sewn together using a sewing machine (Memory Craft 6500 P, Janome, Hachioji, Tokyo) to create the actuator array structure for the sealed actuators to slot into. If the pouches are sewn one on top of each other, elongation actuators are created. If the pouches are sewn along the base onto a strain-limiting inextensible layer, the bending actuators are created, as seen in Fig. 2f. Finally, the manufacturing procedure for the multi-DOF continuum actuators, as seen in Supplementary Video 4, is discussed in Supplementary Materials.

## Constitutive Material Model Fitting of Fabrics and Textiles

**Figure 3:**
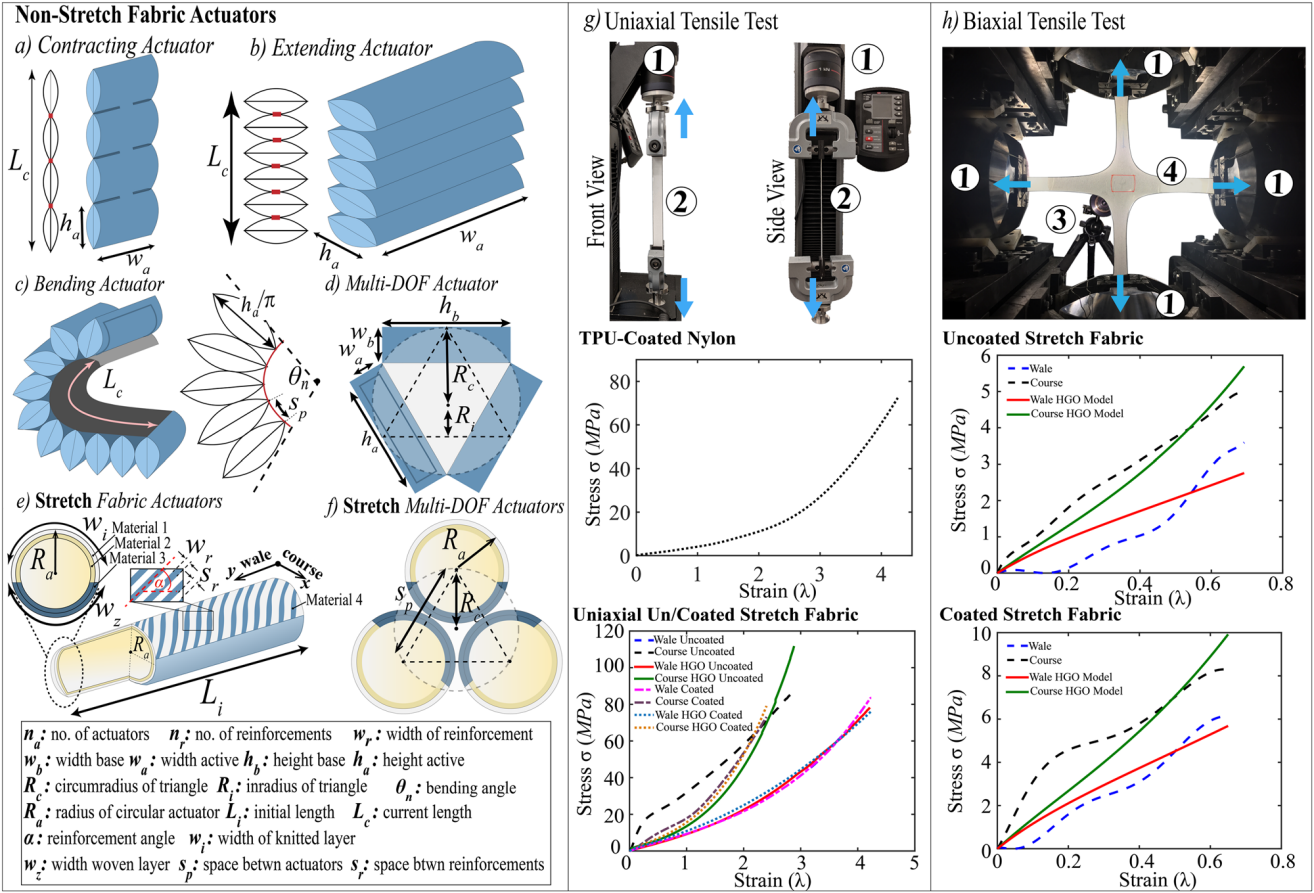
Geometrical parameters for (**a**) woven contracting actuator, (**b**) woven extending actuator, (**c**) woven bending actuator, (**d**) woven multi-DOF actuator, (**e**) FRTA actuators, and (**f**) FRTA multi-DOF actuators. (**g**) Uniaxial tensile test of woven TPU-coated and knitted stretch fabric. (**h**) Biaxial Tensile test of bidirectional knitted stretch fabric.

We try to identify the appropriate material model parameters for the different textiles and fabrics we use as a precursor for the proposed FEM models. In Supplementary Materials, we further described the geometrical parameters, as shown in Fig. 3a–f, and experimental procedure for characterizing the different woven non-stretch and knitted stretch fabrics using uniaxial and/or biaxial universal tensile testing machines, as shown in Fig. 3g,h. We note that the material properties of the TPU-coated materials are within the elastic range while the properties of the knit stretch fabric is considered as an anisotropic hyperelastic material.

Previous FEM-based soft robot modeling work has focused on isotropic elastomeric materials^7,77^. Material properties of these actuators and robots were captured using Arruda-Boyce, Van-der-Waals, Mooney-Rivlin and Neo-Hookean models for smaller strains^7^, and Ogden, Yeoh, and higher-polynomial models for larger hyperelastic strains^78^. However, there have been only one preliminary example of computationally modeling the behavior of knit FSPAs^9^. In this work, we further model the behavior of multi-layered, multi-material (made of woven fabric reinforcements and a knit fabric shell) FRTAs. Some of the constitutive models included in ABAQUS (Simulia, Dassault Systemes) to model anisotropic models include generalized Fung and Holzapfel-Gasser-Ogden (HGO) models.

The material properties of the TPU-coated nylon are within the elastic range, and the Young’s modulus and Poisson’s ratio are calculated as E = 498 $MPa$ and 0.35 using a uniaxial tensile test as seen in Fig. 3g. The inextensible fabric layer used to hold the actuators in the actuator array has the properties E = 305 $MPa$, v = 0.35 and the properties used for the PLA connector caps (E = 3600 *MPa*, v = 0.3). All the components are modeled using shell explicit quadratic tetrahedral elements (C3D10M).

### Anisotropic material model of bi-directional textile materials

The anisotropic hyperelastic properties are evaluated with the HGO continuum model^79^. A non-linear regression model (Limited-memory BFGS^77^) was used to fit material data against HGO hyperelastic strain energy function (see Supplementary Materials for more details). The strain energy equation of the HGO model is as shown below:

1 $$U={C}_{10}({\bar{I}}_{1}-3)+\frac{1}{D}\cdot (\frac{{({J}^{el})}^{2}-1}{2}-ln{J}^{el})+\frac{{k}_{1}}{2{k}_{2}}\mathop{\sum }\limits_{\alpha \mathrm{=1}}^{N}{e}^{{k}_{2}{\bar{E}}_{\alpha }^{2}}-\mathrm{1,}$$

2 $${\bar{E}}_{\alpha }=\kappa ({\bar{I}}_{1}-\mathrm{3)}+\mathrm{(1}-3\kappa )({\bar{I}}_{\alpha \alpha }-\mathrm{1),}$$

where ${C}_{10},D,{k}_{1},{k}_{2},$ and $\kappa$ are the five temperature-dependent material parameters. $N$ is the number of families of fibers ($N\le 3$); ${\bar{I}}_{1}$ is the first invariant of the Cauchy-Green tensor, ${\bar{I}}_{\mathrm{4,6}}$ are the invariants that represent the preferred directions for the fibers contributing to the strain-energy function. If $\kappa$ ($0\le \kappa \le \frac{1}{3}$) is close to $0$, it means the fibers are in the direction of $\theta$ (the course direction); if $\kappa$ is close to $\mathrm{1/3}$, it means the fibers are dispersed and the material would be considered isotropic.

The material fitting tool allows the user to set the poisson ratio, boundary conditions and initial parameters for the material parameters (${C}_{10},D,{k}_{1},{k}_{2},$ and $\kappa$) and the experimental equibiaxial testing data. The Cauchy stress (${\sigma }_{\theta \theta }$, ${\sigma }_{zz}$) is in the course and wale directions. A least-squares fit for the stress-strain equations of both directions is used:

3 $$\chi =\mathop{\sum }\limits_{i\mathrm{=1}}^{n}[({\sigma }_{\theta \theta }-{\sigma }_{\theta \theta }^{model}{)}_{i}^{2}+{({\sigma }_{zz}-{\sigma }_{zz}^{model})}_{i}^{2}\mathrm{]}.$$

The material fitting toolkit also allows the use of multiple optimziation algorithms, such as Nelder-Mead, Powell, CG, L-BFGS-B, COBYLA, and SLSQP, given by the SciPy optimization function^77^. For every iteration, the coefficient of determination ${R}^{2}$ and root mean square of the reduced chi-square $\varepsilon$ were used against the material testing data for the next optimization loop. For the equibiaxial protocol^80^, results were considered acceptable for ${R}^{2}\, > \,0.8$ and $\varepsilon \, > \,0.25$.

After optimization using this scheme, the HGO model is used to fit four tensile testing data sets, two equibiaxial and two uniaxial, as seen in Fig. 3g,h. The same stretch fabric was used for all tests, one set was coated with a TPU layer to aid bonding and air impermeability and another set was not coated with a TPU layer.

For the uncoated uniaxial test, the parameters were identified as C_10_  =  $1.156$, ${k}_{1}$ = $0.0925$, ${k}_{2}$ = $0.0$, $\alpha$ = $0.321$ and $\kappa$ = $0.0$ (the ${R}^{2}\,=\,0.76$ and $\varepsilon \,=\,0.28$). For the coated uniaxial test, the parameters were identified as C_10_ = $1.0$, ${k}_{1}$ = $0.163$, ${k}_{2}$ = $0.0$, $\alpha \,=\,1.93\times {10}^{-12}$ and $\kappa \,=\,0.133$ (the ${R}^{2}\,=\,0.97$ and $\varepsilon \,=\,0.14$).

For the uncoated equibiaxial test, the parameters were identified as C_10_ = $$0.503$$, $${k}_{1}$$ = $$0.138$$, $${k}_{2}$$ = $$0.0$$, $$\alpha $$$$\,=\,0.0$$ and $$\kappa $$ = $$0.0$$, with a resultant $${R}^{2}\,=\,0.88$$ and $$\varepsilon \,=\,0.22$$. For the coated equibiaxial test, the parameters were C_10_ = $$1.098$$, $${k}_{1}$$ = $$0.225$$, $${k}_{2}$$ = $$4.05e-10$$, $$\alpha $$ = $$0.0$$ and $$\kappa $$ = $$2.087\times {10}^{-10}$$ with a resultant $${R}^{2}\,=\,0.8$$ and $$\varepsilon \,=\,0.22$$.

## Modeling of fabric-based actuators using FEM(iv)

In this work, computational FEM models are created to capture the performance of the various fabric-based actuators. The effects of their geometrical parameters, highlighted in Fig. 3a–f) and Supplementary Materials, are studied for blocked force and displacement tests using the computational FEM modeling tool written in Python 2.7, for ABAQUS/Explicit (Simulia, Dassault Systemes). The modeling tool is capable of automating the process of creating the part, meshing, and applying boundary conditions based on the user-defined parameters. Computational models enable rapid design iterations prior to actual fabrication of the prototypes.

ABAQUS/Explicit is used to capture the short dynamic response times observed among different types of fabric actuators. ABAQUS/Explicit is also capable providing both dynamic and quasi-static solutions for blocked force and displacement tests of the different types of actuators. In order to perform quasi-static simulations, the explicit solution would need to be accelerated while still maintaining its dynamic equilibrium^81^. To maintain dynamic equilibrium the loading rate of the analysis needs to be 1% of the speed of the stress wave of the material^81^. To monitor dynamic equilibrium, the total kinetic (KE) and internal (IE) energy of the entire system are monitored to ensure that KE does not exceed 5% of total IE^81^.

The airflow dynamics within the chambers is disregarded and modeled as pressure equally applied on the actuators’ internal surfaces. The pressure is designed as a smooth ramp step to the desired value. Gravity is not considered in the models due to the lightweight nature of the actuators.

In order to measure the displacement of the actuators, passive reflective markers are attached on the fabric actuators during experiments. For measuring bending angle, three markers are distributed evenly along the length of the actuator. For measuring displacements in the three axes, markers are placed at the distal and proximal ends of the actuators. A motion capture system (Optitrack Prime 13 W, NaturalPoint Inc., Corvallis, OR) is used for experiments, and each experiment was repeated three times. For measuring the payload of the actuators, we denoted the experimental setup in Supplementary Fig. 3.

### FEM models for woven fabric actuators

**Figure 4:**
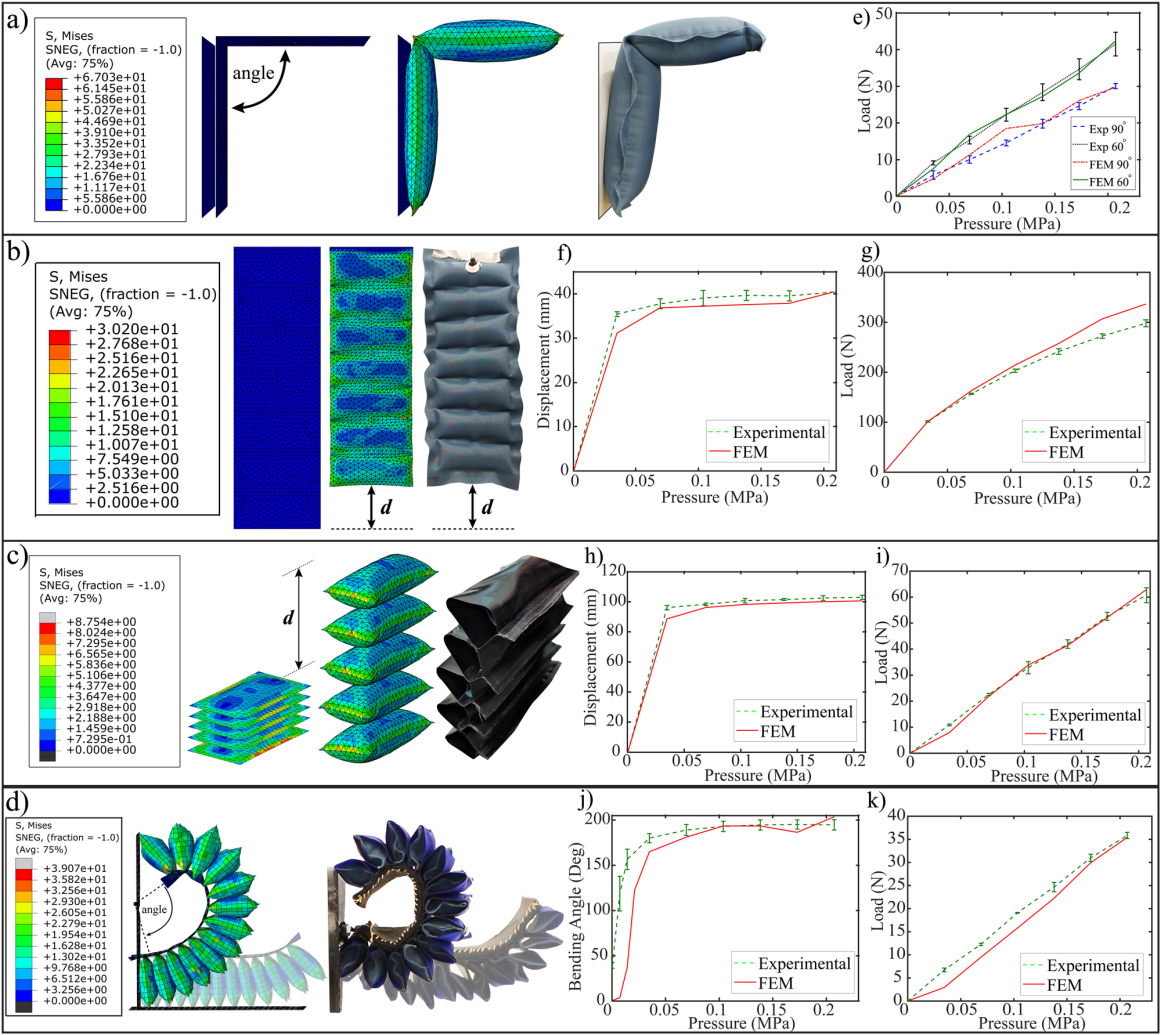
Woven non-stretch fabric actuators: FEM v.s. experimental results for (**a**) stiffening actuator, (**b**) contracting actuator, (**c**) elongating actuator, and (**d**) bending actuator. (**e**) Straightening actuator: load v.s. pressure. (**f**) Contracting actuator: displacement v.s. pressure. (**g**) Contracting actuator: load v.s. pressure. (**h**) Elongating actuator: displacement v.s. pressure. (**i**) Elongating actuator: load v.s. pressure. (**j**) Bending actuator: bending angle v.s. pressure. (**k**) Bending actuator: load v.s. pressure.

Computational models for different woven non-stretch fabric actuators including the stiffening, contracting, elongating, bending are developed. Figure 4a–d and Supplementary Video 1, shows the Von Mises stress contour plots obtained from the FEM simulations, along with the experimental results of the pressurized actuators at the corresponding input pressure. The force output (payload) and displacement (bending angle, extension, or contraction) are measured at small pressure increments of 0.034 $MPa$ until a safe operating pressure of 0.206 $MPa$.

The stiffening actuators are used for applications that require an extension motion, such as assisting the knee, wrist, elbow, and finger joints. Comparison test between the FEM model and experimental prototype is conducted for an actuator with an ${w}_{a}$ = 65 *mm* and an ${L}_{i}$ = 240 *mm*. For the block force experiments, the actuator was positioned at a desired bending angle of 60° and 90°. The simulation shows similar performance for the ${60}^{\circ }$ angle with an RMSE of $1.08N$, and for the ${90}^{\circ }$ angle with an RMSE of $1.71N$, as shown in Fig. 4e.

The contracting actuators are used for applications that require pulling or contracting. The geometrical parameters of the actuator used include, ${n}_{a}$ = $7$, ${L}_{i}$ = 200 *mm*, ${w}_{a}$ = 60 *mm*, and ${h}_{a}$ = 22.86 *mm*, and with a centralized air passage with a width of 5mm. For the displacement and blocked force tests, the contracting length ($d$) and pulling contraction force were measured, respectively. For the displacement test, a maximum displacement error of $\mathrm{13.84 \% }$ and an RMSE error of $2.06mm$ are noticed, as seen in Fig. 4f. The blocked force tests for the modules are modeled with both the top and bottom end-plate faces fixed in all directions (encastre) when under external pressure load. The simulation predicts the force well up to around 0.17 $MPa$, after which the simulation shows slightly higher force readings than the experimental results possibly due to slight air leakage in the prototype because of the material being stretched because of pulling forces of around 270 $N$. The RSME of $21.02N$ and a maximum force error of $\mathrm{11.45 \% }$ is noticed, as seen in Fig. 4g.

The elongating actuators’ geometrical parameters include, active width (${w}_{a}$) of 62 *mm* and active height (${h}_{a}$) of $31mm$. Experimental data is gathered for a stack of five actuators (${n}_{a}\,=\,5$). The free displacement is compared to simulation results as seen in Fig. 4h. The maximum displacement error of $\mathrm{8.52 \% }$ and the RMSE error of $3.46mm$ are observed. For the blocked force test seen in Fig. 4i comparing the experiment and simulation, an RMSE of $1.49N$ is observed. Both free displacement and blocked force simulations show a good prediction of the experimental results.

The bending actuators, designed for various flexion applications are tested for bending angle and blocked force, as shown in Fig. 4d. For the displacement and blocked force tests, actuators with $n$ = $13$, ${w}_{a}$ = 41 *mm*, and ${h}_{a}$ = $30mm$ were used. The results are shown in Fig. 4j and k. For this test, a vertical plate is designed to limit the distal end of actuator from further curling inwards during inflation, to maintain bending angles at around 200°, for ease of monitoring and calculating the bending angles. It is noticed that the bending actuator prototype has an initial bending angle because the fittings on each actuator create an initial stiffness. However, at around 30–40% of the simulation the FEM model catches up the experimental data where we see results closely match between the simulation and actual experiments. For the blocked force test, the FEM simulation catches up to the experimental data at around the 60–65% of the simulation. Both present similar payload outputs with a RSME of $2.39N$.

### FEM models for knit bi-directional stretch fabric actuators

**Figure 5:**
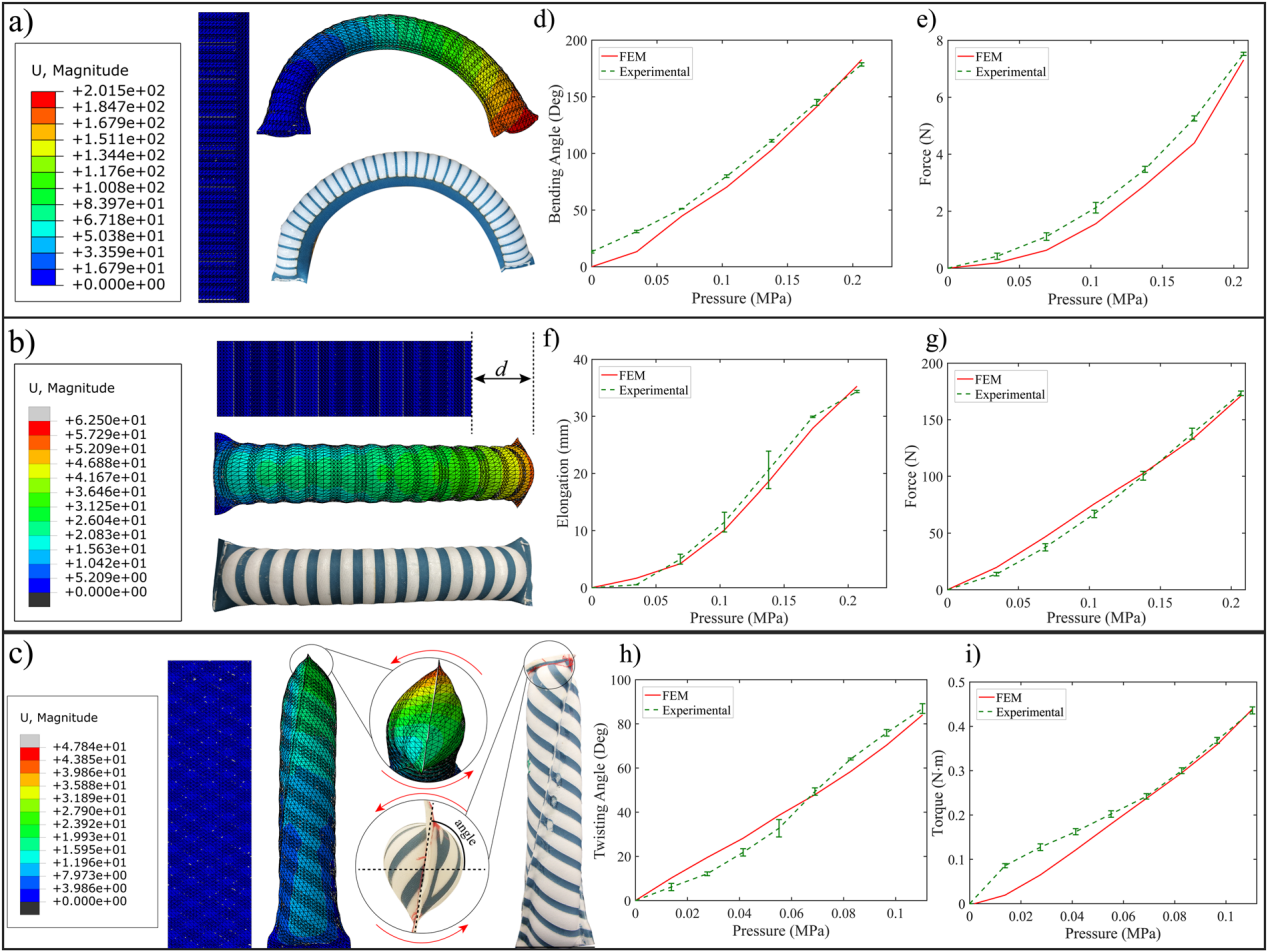
Knitted stretch fabric actuators: FEM v.s. experimental results for (**a**) bending actuator, (**b**) elongating actuator, and (**c**) twisting actuator. (**d**) Bending actuator: displacement v.s. pressure. (**e**) Bending actuator: load v.s. pressure. (**f**) Elongating actuator: displacement v.s. pressure. (**g**) Elongating actuator: load v.s. pressure. (**h**) Twisting actuator: twisting angle v.s. pressure. (**i**) Twisting actuator: torque v.s. pressure.

Computational models are created to study the effects of the fabric reinforcement on the motion profile of different knit stretch FRTAs. Figure 5a-c and Supplementary Video 2 shows the displacement contour plots obtained from the FEM simulations compared with the experimental images of the pressurized actuators at the corresponding pressure values. The main geometrical parameters studied are the number of reinforcements ($n$) and angle of the fabric reinforcements ($\alpha$), as seen in Supplementary Fig. 2. The force output (payload or torque) and displacement (bending angle, twisting angle, or elongation) are measured at small pressure increments for both the FEM simulations and experiments.

The bending FRTAs were tested for bending angles (Fig. 5d and blocked forces (Fig. 5e). For both tests, the actuator’s geometrical parameters used were ${L}_{i}=155\,mm$, ${w}_{r}=1.5\,mm$, $\alpha {=0}^{\circ }$, ${w}_{i}=40\,mm$, ${w}_{z}=14\,mm$, and ${n}_{r}=35$. We notice for the bending angle test, the results are closely matched between simulation and actual experiments with an RMSE of ${10.16}^{\circ }$. For the bending FRTA prototype, there was an initial bending angle because the prototype had a small initial stiffness. For the load test, the experimental results followed the same trend as the simulation, with an RMSE of $0.4939N$.

The elongating FRTAs were tested for displacements (Fig. 5f and blocked forces (Fig. 5g). The actuator’s geometrical parameters used for both tests were ${L}_{i}=155\,mm$, ${w}_{r}=6.0\,mm$, $\alpha {=0}^{\circ }$, ${w}_{i}=46\,mm$, ${w}_{z}=0.0\,mm$, and ${n}_{r}=15$. From the displacement graph, Fig. 5f, we notice that the FEM model matches the experimental data with an RMSE of $1.36\,mm$. For the blocked force graph, Fig. 5g, the FEM model predicts the payload of the actuator very closely with an RMSE of $5.81N$. For the elongating FRTA, the fabric reinforcements convert the radial expansion to axial extension, therefore a higher the number of reinforcement leads to less radial expansion and more elongation.

The twisting FRTA models were experimentally validated for twisting angles and torque capability, as shown in Fig. 5h, i. The actuator was inflated to 0.11 $MPa$ with increments of 0.014 $MPa$, which was selected as a safe maximum input pressure in order to prevent any prominent radial expansion that might cause actuator failure. The actuator’s geometrical parameters were *L*_*i*_ = 155 mm, *w*_*r*_ = 5.0 mm, *α* =−30°, *w*_*i*_ = 46 mm, *w*_*z*_ = 0.0 mm, and *n*_*r*_ = 16. The FEM model predicts the twisting angle of the actuator well, with an RMSE of 4.94°. Based on previous work with fiber reinforcements^82^, the twisting capability of the actuator, clockwise or counterclockwise (|*α*|), improves gradually from 0 to 30° and then reduces until $|\alpha {\mathrm{|=90}}^{\circ }$, where the reinforcements are symmetric preventing the actuator from twisting and promoting just radial expansion. For the blocked torque capability, the FEM model predicts lower torque values up until around 50–60% of the simulation, where the payload of the experimental results match the simulation results very closely with an RMSE of 0.0352 $N\cdot m$.

## Case study of FSPAs in wearable applications

**Figure 6:**
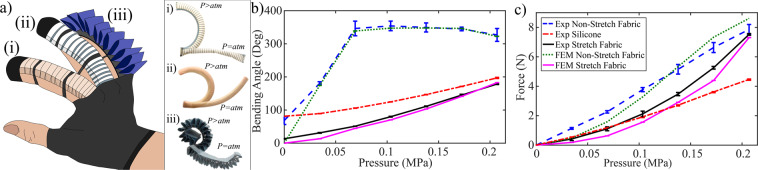
Comparisons of soft actuators for finger assistance. (**a**) [Left] Highlights the different actuators worn on a user’s hand. [Right] The different types of actuators deflated and inflated. i) Knitted fabric actuator, (ii) elastomeric actuator, and (iii) woven fabric actuator. (**b**) Angle v.s. pressure for three different actuators. c) Load v.s. pressure for three different actuators.

One of the popular assistive/rehabilitative application for SPAs has been soft robotic gloves for patients suffering with reduced hand functionality^8–10^. We demonstrate the capabilities of the woven FSPAs and the knit FRTAs in comparison with the existing fiber-reinforced elastomeric actuators^10^, for finger flexion, as seen in Fig. 6a. According to literature, the requirements for flexion of the human index finger^10^ includes a bending angle of at least 160° and a distal tip force of approximately 7.3$N$.

Computational models of the FSPAs are modeled using the same geometrical parameters of the fiber-reinforced elastomeric actuator^10^, in order assess the design before fabrication. The common geometrical parameters for the actuators are ${R}_{a}=10\,mm$, ${w}_{z}=10\,mm$, ${L}_{i}=155\,mm$. The woven non-stretch actuators has ${n}_{a}=19$, ${s}_{p}=9\,mm$ and ${w}_{a}=20\,mm$ and ${h}_{a}=20\,mm$. The knitted stretch actuators has ${n}_{r}=35$, ${w}_{r}=1.35\,mm$, and ${s}_{p}=1.5\,mm$. The FEM models are experimentally validated for bending angles and tip force payloads, while inflating the specimens up to 0.206 $MPa$ with a small pressure increment of 0.034 $MPa$, as seen in Fig. 6b,c. Both the fabric-based actuator FEM models meet the motion and force requirements. The distal tip forces of the fabric-actuators obtained both through the FEM simulation are experimentally validated, resulting in an RMSE of 0.59 $N$ and 0.49 $N$, for the woven FSPA and knit FRTA respectively. Both the experimental and FEM model data demonstrated similar bending behavior with an RMSE of 26.2° for the woven FSPA and 10.16° knit FRTA as seen in Fig. 6b. The woven FSPA prototype displays an initial bending angle because of the stiffness due to plastic fittings assigned to each actuator.

From Fig. 6a and Supplementary Video 3, we compare the bending angles and distal tip forces of the three actuators together. The woven FSPA instantly bends and curls when pressurized and reaches its maximum bending angle, at 0.069 $MPa$, which is approximately 1.7 × larger than the silicone and stretch fabric actuators’ bending angles. Therefore, this actuator reaches it’s maximum bending angle the quickest. On the other hand, the FRTA and fiber-reinforced actuators steadily reach similar maximum bending angles at 0.206 $MPa$. The silicone actuator also display a slight initial bending angle because the initial stiffness exhibited by the material’s stiffness, with a Hardness Shore $28A$. As seen in Fig. 6c, the fabric-based actuators demonstrate approximately a 1.71 × higher payload at $0.206MPa$, meeting the distal force requirements for the task at a lower operating pressure. The silicone actuator needs to be pressurized till 0.275 $MPa$ to meet the desired tip force. In terms of weight, the silicone, woven FSPA and knit FRTA actuators are 37.5 $g$, 82.5 $g$, and 9.7 $g$ respectively (with pneumatic fittings). The additional weight of the woven non-stretch fabric is due to the pneumatic fittings on each actuator in the array. Therefore, the FRTA actuators show the highest force-to-weight ratio in comparison to the other actuators. A prototype of the assistive wearable glove made of the FRTAs is presented in Supplementary Fig. 2.

We further characterized these three actuators for their frequency response and efficiency, as seen in Supplementary Materials. For the frequency test, we noticed that the fiber-reinforced elastomeric actuator, knit fabric-reinforced textile actuator, and woven fabric FSPA had the frequency response of 2 $Hz$, 0.7 $Hz$, and 0.45 $Hz$, respectively. This is highlighted in Supplementary Fig. 4 and Supplementary Video 5. We also analyzed the external energy interactions, based on^83,84^, of these actuators as seen in Supplementary Figs. 5 and 6. From the overall efficiency tests, the elastomeric actuator, woven FSPA, and knit FRTA have maximum efficiencies of 0.785% at 0.05 kg, 0.287% at 0.1 kg, and 0.26% at 0.2 kg, summarized in Supplementary Table 4.

## Discussion and Conclusion

In this paper, we explored the combination of various textiles to mechanically program actuators to perform different motion profiles, while still being lightweight, compliant, and safe. We introduced two main classes of versatile fabric-based soft pneumatic actuators, the woven non-stretch fabric actuators and the knit fabric reinforced textiles actuators. The woven fabric actuators used the interaction of multiple actuators arranged in different array fashions to create various motion profiles. On the other hand, the FRTAs perform a combination of motions by utilizing the interaction of the woven fabric-reinforcements along the length of the mechanically anisotropic knit high-stretch fabric body. Both types of FSPAs demonstrated the potential to deliver significant blocked forces and displacements in comparison to the conventional fiber-reinforced elastomeric actuators without introducing any mechanical instability, while still being highly wearable, lightweight, compliant, and safe. However, preliminary frequency testing has shown us that due to the fabrics’ pliability and thin-walled material properties, it shows a lower maximum operable frequency in comparison to the fiber-reinforced elastomeric actuators. From the preliminary efficiency tests, the relatively thick walled fiber-reinforced elastomeric actuators show a higher efficiency when lower work is done, but all three actuators show similar efficiency at higher work.

To improve the time-consuming limitations with manufacturing often seen in SPAs, we presented rapid and low-cost 2D manufacturing methods to develop these FSPAs using commercially available fabrics. These external fabric reinforcements that create a meta-material frame are designed accurately with any varying geometrical parameters, and perfectly aligned around the anisotropic textile body of the FRTAs. The manufacturing method can be easily scaled and can produce even more complex geometries to benefit any assistive and rehabilitative tasks.

We also comprehensively studied and mechanically characterized the various fabrics used to generate non-linear constitutive material models for large deformations based on the HGO form^79^ using bi-directional stress and strain data representing the mechanical anisotropy of the material. We implemented an extensive library of experimentally validated, FEM models for FSPAs (4 woven and 3 knit FSPAs). These models can be utilized as design tools for the users to vary the actuator’s geometrical parameters and materials, in order to predict the mechanical response of the actuators to internal quasi-static and dynamic pressure, as well as external contact. This will benchmark the design criteria for developing scalable and customizable FSPAs based on the articulation performance requirement and desired payload prior to fabrication.

We aim to add the capabilities of distributed, embedded fabric sensing technologies, to monitor the articulation of the actuators and the interaction with the users and environment. Future work will also investigate the design of the actuators with user ergonomic considerations. Some key considerations will include selection of attachment points on the body to distribute the load along with various feedback/feedforward control strategies. Further exploration of the dynamic and time-dependent responses and dynamic hysteresis, of the actuators would need to be evaluated for various pressurization patterns. Future work will include more in-depth and comprehensive frequency and efficiency testing. For the frequency test, more variations of the duty cycle between pressurization and venting will be tested. The overall frequency of the FSPAs can also be improved by increasing the inlet size of the connectors, to improve the flow in and out of the actuator. For the efficiency test, the initial volume of the actuators will be accounted as well as the efficiency of the actuators during dynamic motion. The future models will also allow the users to evaluate and optimize the actuators based on efficiency and volume considerations that tie into on-board portability considerations. Finally, future research will also include analytical models of the non-linear behaviors of the fabrics at large deformations using the FEM models in this work to provide a baseline necessary for analytical characterization of these actuators.

## Supplementary information

Supplementary Video 1.

Supplementary Video 2.

Supplementary Video 3.

Supplementary Video 4.

Supplementary Video 5.

Supplementary Materials
