# Author Correction: Design and Computational Modeling of Fabric Soft Pneumatic Actuators for Wearable Assistive Devices

**DOI:** 10.1038/s41598-020-69094-9

**Published:** 2020-07-16

**Authors:** Pham Huy Nguyen, Wenlong Zhang

**Affiliations:** The Polytechnic School, Ira A. Fulton Schools of Engineering, Arizona State University, Mesa, AZ 85212 USA

Correction to: *Scientific Reports* 10.1038/s41598-020-65003-2, published online 15 June 2020

This Article contains errors in Reference 13 which was incorrectly given as:

Nassour, J. & Hamker, F. Enfolded textile actuator for soft wearable robots. In *2018 7th IEEE International Conference on Biomedical Robotics and Biomechatronics (Biorob)* (2020).

The correct reference is listed below as ref 1:

1. Nassour, J. & Hamker, F. Enfolded Textile Actuator for Soft Wearable Robots. In *2019 IEEE International Conference on Cyborg and Bionic Systems (CBS),* 10.1109/CBS46900.2019.9114425 (2019).

