# Supplementary material for: Design and Computational Modeling of Fabric Soft Pneumatic Actuators for Wearable Assistive Devices
Source: Sci Rep. 2020 Jun 15;10:9638. doi: 10.1038/s41598-020-65003-2 (PMC7295994; doi:10.1038/s41598-020-65003-2)
Supplement: Supplementary file 6 — Supplementary Materials [file 41598_2020_65003_MOESM6_ESM.pdf]

Design and Computational Modeling of Fabric Soft Pneumatic Actuators for Wearable Assistive Devices

Pham Huy Nguyen[1] **and Wenlong Zhang**[1,*]

[1]The Polytechnic School, Ira A. Fulton Schools of Engineering, Arizona State University, Mesa, AZ 85212, USA
[*]corresponding.wenlong.zhang@asu.edu

Supplementary Materials

Types of Actuators and Assistive Tasks

Table 1. The type of motions required in biomedical rehabilitative and assistive devices, the targeted applications, and the possible fabric soft pneumatic actuators (FSPAs) that can assist.

Type of Motion	Targeted Applications	Possible Fabric Soft Pneumatic Actuator (FSPAs)
**Bending**	Flexion Devices (knee, wrist, elbow, finger, etc.)	Woven FSPAs, Textile Actuators, Fabric-Reinforced Textile Actuators
**Stiffening**	Extension Devices (knee, wrist, elbow, finger, etc.)	Woven FSPAs, Textile Actuators, Fabric-Reinforced Textile Actuators
**Twisting**	Supination/Pronation Devices (forearm, ankle/foot, etc.)	Textile Actuators, Fabric-Reinforced Textile Actuators
**Contracting/Elongating**	Devices that mimic muscle functionality (biceps and triceps contraction, heart muscles, etc.)	Woven Peano-FSPAs, Fabric-Reinforced Textile Actuators
**Multiple Degrees-of-Freedom**	Devices for the neck, the spine, the shoulder, etc.	Mutli-DOF Woven FSPAs, Mutli-DOF Fabric-Reinforced Textile Actuators

Fabrication of the Multi-DOF FSPAs

To manufacture the multi-DOF continuum actuators, 3D-printed acrylonitrile butadiene styrene (ABS) plastic connectors are made. The connectors include tiny holes so the user can manually stitch, with Kevlar thread (8800K44, McMaster-Carr, Elmhurst, IL), the ends of the actuators to the top and bottom connector plates. The actuators are stitched in an equilateral triangle prism fashion to create the multi-DOF continuum actuators.

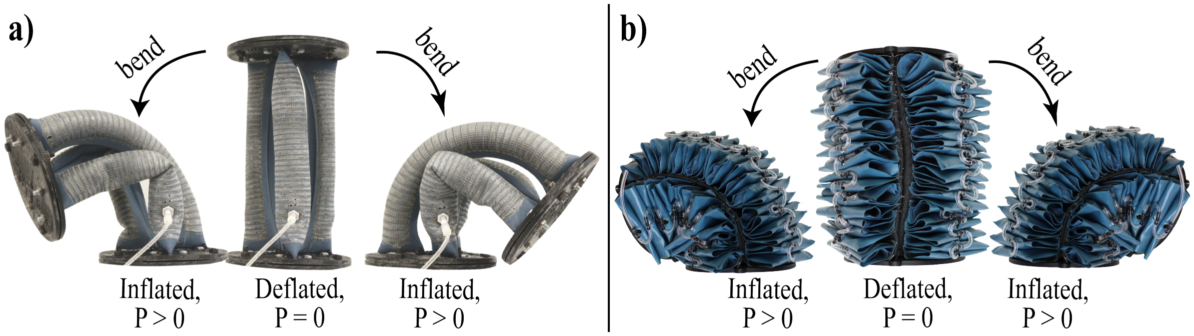

Figure 1. The Multi-DOF continuum actuators (a) Knitted Stretch Fabric Multi-DOF Actuator (b) Woven Fabric Multi-DOF Actuator

Geometrical Parameters of Actuators

Geometrical Parameters of Woven Fabric Actuators

The geometrical parameters of the woven non-stretch fabric actuators are summarized in Figs. 3a)-d). For these actuators, multiple motion profiles are created by combining actuators in various array fashions with or without a strain-limiting layer, to create bending, contracting, elongating, straightening, and multi-DOF actuators.

The sewn connection lines between each actuator in the array are highlighted with red lines in Figs. 3a)-c). The main geometrical parameters include the initial and current length during inflation (L_i and L_c), the active height and width of the actuator units during inflation (w_a and h_a), and the number of actuators in the actuator array (n_a). The assumption taken here is that when each actuator inflates, it creates a circular cross-section with a diameter of h_a/π, seen in Fig. 3a)-c). Thus the n_a and h_a determine the current length (L_c) of the contracting and extending actuator arrays and the bending capability (θ_n) of the bending actuator array, as calculated below:

$$L_c = \frac{h_a}{\pi} \cdot n_a, \tag{1}$$

$$\theta_n = 2n_a \cdot sin^{-1}(1 - \frac{s_p \cdot \pi}{2h_a}). \tag{2}$$

The ratio (r) between the active width and height (w_a and h_a) contributes to the stability of the actuator array. If the w_a is smaller than h_a, the contact area between the consecutive actuators will be smaller, leading to possible torsional motion and reduction in the force distribution capability of the array.

For the bending actuator, the spacing between the actuators (s_p) as seen in Fig. 3b, is limited by how close the actuators can be sewn together. It also controls the number of actuators (n) that can be fit within the initial length of the actuator (L_i). Finally, for the woven multiple-DOF actuator (as seen in Fig. 3d), R_c (the cross-sectional radius of the system) constrains the size of the individual actuators (w_a and h_a). The ratio (r), spacing (s_p), and active width (w_a) can be found as follows:

$$r = \frac{w_a}{h_a}, \tag{3}$$

$$s_p = \frac{(L_i - 2 \cdot (L_i/n_a))}{n_a}, \tag{4}$$

$$w_a = R_c/(\frac{1}{\sqrt{12}} + \frac{1}{r}). \tag{5}$$

Geometrical Parameters of Stretch Fabric Actuators

The geometrical parameters of the FRTAs study the effects of using woven fabric reinforcements to generate complex motions, including bending, twisting, elongating, and a combination of combination of multiple of these actuators can create knitted multiple DOF actuators as well. This is highlighted in Figs. 3e) and f).

As seen in Fig. 3e), Material 1 is the knitted stretch fabric, Material 2 is the internal TPU bladder material, Material 3 is the material for the strain-limiting layer, and Material 4 is the woven fabric reinforcements. In this work, Materials 3 and 4 are the same woven TPU-coated nylon material. The three main geometrical parameters are the number of fabric reinforcements (n_r), thickness of the reinforcements (s_r), and the angle of reinforcements (α).

The reinforcement angle (α) controls the motion of the actuator during inflation causing it to either elongate, radially expand, or twist along the central axis. The number of fabric reinforcements (n_r) and thickness of the reinforcements (s_r) affect the amount of radial expansion seen in the actuator. The radius of the actuator (R_a) is defined by the width of the knitted stretch fabric (w_i) and inelastic woven strain-limiting layer (w_z). This w_z is larger than zero for cases where a straining limiting layer is desired, in the case of a knitted bending actuator. Thus, w_z is zero for extending and twisting actuators. For the stretch multi-DOF system, s_p determines the distance between the center points of the actuators placed in an equilateral triangle formation. These relationships can be seen in Eq. (7) below:

$$R_a = \frac{(w_z + w_i)}{2\pi} \tag{6}$$

$$n_r = \frac{L_i \cdot cos(\alpha)}{w_r + sr}. \tag{7}$$

In this work, actuators with only a single family of reinforcements (fabric) are used. Pure elongation (when $\alpha = 0°$) can be achieved with FRTAs using just that single family of reinforcements, which is accurately laser-cut and aligned to laminate over the knitted textile. This reduces any variability during fabrication. The thickness of the fabric reinforcements (s_r) is also adjustable by varying the fabric material therefore able to further reduce any unwanted twisting motion. To create a pure twisting FRTA, the angle of the reinforcements (α) has to be larger (clockwise twist) or smaller (counterclockwise twist) than 0°. When $\alpha = \pm\ 30°$ the best achievable twisting motion is seen.

Characterization of Fabric Materials for Modeling

We first describe the experimental procedures for characterizing different woven non-stretch and knitted stretch fabrics. Most high-strength fabrics are categorized using a dernier number (D) that describes the thickness of the fibers used.
We mechanically characterize the properties of the non-stretch and the high-stretch textiles by conducting uniaxial tension tests using a universal testing machine (UTM Instron 5944, Instron Corp., High Wycombe, United Kingdom). The knitted stretch textiles with different bidirectional stretch were characterized using a biaxial testing machine (MTS Biaxial/Torsional test system) in using equibiaxial tension tests, as seen in Figs. 3g) and h).

Table 2. Material Properties of TPU Coated Nylon Fabrics

Material	*Density (kg/m^3)*	*Seal Strength (N)*	*Burst Strength (MPa)*
Rockywoods 200D	840.00	168.35	0.53±0.04
Outdoor Oxford 200D	757.58	183.68	0.48±0.03
Ripstop 200D	758.62	192.19	0.40±0.017
Seatle Diamond 200D	892.86	164.75	0.36±0.026
DIY Packraft 400D	982.46	155.32	0.20±0.026
DIY Packraft 1000D	1000.00	236.17	0.11±0.02
Taffeta 70D	700.00	150.59	0.048±0.008

Non-stretch TPU-coated Fabrics

TPU-coated nylon fabrics with different dernier values (from 70D to 1000D) are summarized in Supplementary Table 2 based on their seal and burst strength. To execute the peel test, the ASTM F88/F88M-15 protocol with a uniaxial tensile testing machine was followed. Two pieces of TPU-coated nylon fabrics were cut into $25.4\times76mm$ and fin-sealed with a $5mm$ width. For the burst test, actuators were created with a dimension of $63.5\times127mm$ and tested to the maximum pressure it could withhold until failure, using the ASTM F2054 protocol. The selected material for this work was a 200D heat-sealable fabric (6607, Rockywoods Fabric, Loveland, CO) had a seal strength of $168.35N$ and a burst strength of 0.53±0.04, as seen in Supplementary Table 2. The stress-strain curve of the material is shown in Fig. 3g).

Bi-directional Stretch Textiles

The mechanical properties of the bi-directional high stretch textiles are tested using both the uniaxial and biaxial tensile testing machines, as seen in Fig. 3h). The high stretch textiles have different stretch properties in two different directions, referred to as wale (in the y-direction) and course (in the x-direction). In this work, the high stretch knitted textile (24350, Darlington Fabrics, Westerly, RI) is made of 17% 210D Denier Spandex fibers and 83% 50 Denier Semi-Dull Nylon.

Uniaxial Testing of Bi-directional Stretch Textile

The properties of the stretch textile were characterized using a uniaxial tensile testing machine. Two types of material samples were used, one was coated with a layer of TPU and the other was uncoated. The layer of TPU is added to increase the air impermeability features of the high-stretch fabric. The ISO-139134-1 standard was used to separately test the wale and course directions of the stretch fabrics. Samples of $50\times150mm$ were used.

In the wale direction, the uncoated sample had more stretch of up to 426.8% at $72.41MPa$ in comparison to the course direction, which was approximately 240.1% at $73.14MPa$. The TPU-coated samples showed an overall stiffness increase in both directions but still follow a similar trend with 422.9% at $83.78MPa$ in the wale direction and 240.1% at $71.01MPa$ in the course direction.

Biaxial Testing of Textile

To further characterize the material properties of the bi-directional high stretch textiles, an equibiaxial test was conducted for both coated and uncoated samples. The samples had a thickness of 0.6mm, arm length of $101.6mm$ and arm width of $25.4mm$. The cruciform square gauge region had sides of a size of $25.4\times25.4mm$. The textiles behaved similarly in both directions in the equibiaxial tests, where as in the uniaxial tests the course direction stretched much more than the wale direction. The uncoated sample had more stretch in both directions at 647% at $4.794MPa$ (course direction) and 647% at $3.332MPa$ (wale direction). The coated sample, on the other hand, had less stretch 509% at $7.231MPa$ (course direction) and 540% at $5.782MPa$ (wale direction), as seen in Fig. 3h).

Anisotropic Material Model of Bi-directional Textile Materials

The anisotropic hyperelastic properties are evaluated with the aniostropic Holzapel-Gasser-Ogden continuum model. The strain energy equation of the HGO model is as shown below:

$$U = C_{10}(\bar{I}_1 - 3) + \frac{1}{D}\cdot(\frac{(J^{el})^2 - 1}{2} - lnJ^{el}) + \frac{k_1}{2k_2}\sum_{\alpha=1}^{N} e^{k_2\bar{E}_\alpha^2} - 1, \tag{8}$$

$$\bar{E}_\alpha = \kappa(\bar{I}_1 - 3) + (1 - 3\kappa)(\bar{I}_{\alpha\alpha} - 1), \tag{9}$$

$$\bar{I}_1 = \lambda_\theta^2 + \lambda_z^2 + \frac{1}{\lambda_\theta^2 + \lambda_z^2}, \tag{10}$$

$$\bar{I}_{4,6} = \lambda_\theta^2 \cos\alpha^2 + \lambda_z^2 \sin\alpha^2, \tag{11}$$

where C_{10}, D, k_1, k_2, and κ are the five temperature-dependent material parameters. N is the number of families of fibers ($N \leq 3$); $\bar{I}_1$ is the first invariant of the Cauchy-Green tensor, $\bar{I}_{4,6}$ are the invariants that represent the preferred directions for the fibers contributing to the strain-energy function. If κ ($0 \leq \kappa \leq \frac{1}{3}$) is close to 0 then that means the fibers are in the direction of θ (the course direction), if κ is close to $1/3$ that means the fibers are dispersed and the material would be considered isotropic. The parameter D is related to the bulk modulus, K_0. Where $D = \frac{2}{K_0}$. The parameter D is set to 0 because the material is modeled as incompressible. The C_{10} parameter is part of the incompressible isotropic model portion of the strain energy equation. It is also known as the neo-Hookean parameter.The set of parameters (k_1 and k_2) is equal when the the fiber families have the same mechanical properties. In this work, we assume the mechanical response of the material is same in the θ. The stretch values (λ_θ, λ_z), strain (ε_θ, ε_z), and Cauchy stress ($\sigma_{\theta\theta}$, σ_{zz}) in the course and wale directions are seen as:

$$\lambda_\theta = 1 + \varepsilon_\theta, \tag{12}$$

$$\lambda_z = 1 + \varepsilon_z, \tag{13}$$

$$\sigma_{\theta\theta} = (\frac{F_\theta}{tw})\lambda_\theta, \tag{14}$$

$$\sigma_{zz} = (\frac{F_z}{tw})\lambda_z, \tag{15}$$

where t and w are the thickness and width of the material, respectively. The deformation gradient and the Cauchy stress tensors (σ) in both directions are given as:

$$F = \begin{bmatrix} \lambda_\theta & 0 & 0 \\ 0 & \lambda_z & 0 \\ 0 & 0 & \frac{1}{\lambda_\theta\lambda_z} \end{bmatrix}, \tag{16}$$

$$\sigma = \begin{bmatrix} \lambda_\theta \frac{\partial U}{\partial \lambda_\theta} \\ \lambda_z \frac{\partial U}{\partial \lambda_z} \\ 0 \end{bmatrix}. \tag{17}$$

The material fitting tool allows the user to set the poisson ratio, boundary conditions and initial parameters for the material parameters (C_{10}, D, k_1, k_2, and κ) and the experimental equibiaxial testing data. A least-squares fit for the stress-strain equations of both directions is used:

$$\chi = \sum_{i=1}^{n}[(\sigma_{\theta\theta} - \sigma_{\theta\theta}^{model})_i^2 + (\sigma_{zz} - \sigma_{zz}^{model})_i^2]. \tag{18}$$

Soft Assistive Wearable Glove

In this section we show a prototype of the soft assistive wearable glove designed using the fabric reinforced textile actuators, introduced in this paper, seen in Supplementary Fig. 2. Future work will include a complete study of the assistive glove for functional grasping, ergonomics, user interaction, and portability.

Experimental Payload Testing Setup

In order to investigate the load performance of the different actuators, we designed experimental tests with all the actuators tested in this work, four woven non-stretch FSPAs and three knit stretch FSPAs. All of the bending and torque payload tests are performed on the a universal testing machine (UTM Instron 5944, Instron Corp., High Wycombe, United Kingdom) and each output is measured at small pressure increments of $0.034 MPa$. Each experiment is repeated three times. The performance characteristics of the FSPAs are highlighted in Fig. 4.

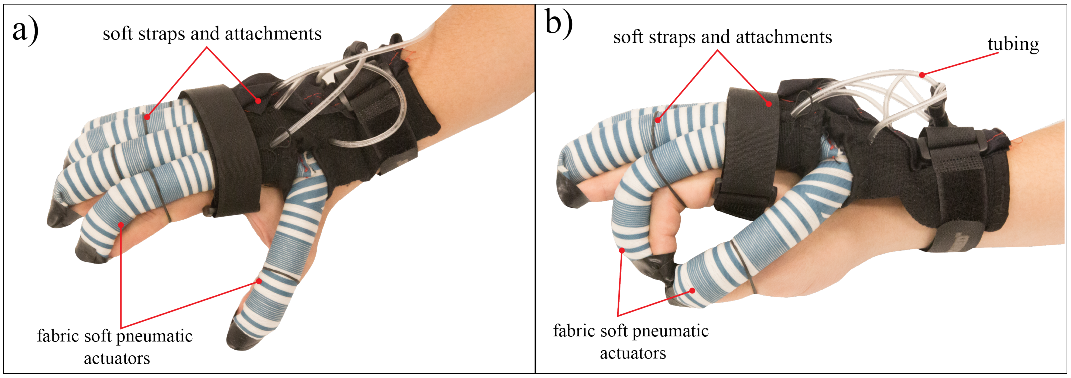

Figure 2. Prototype of the Soft Assistive Wearable Glove. a) Unrestricted open palm of user with the Soft Assistive Wearable Glove. b) Pinch grasp of the user with the Soft Assistive Wearable Glove.

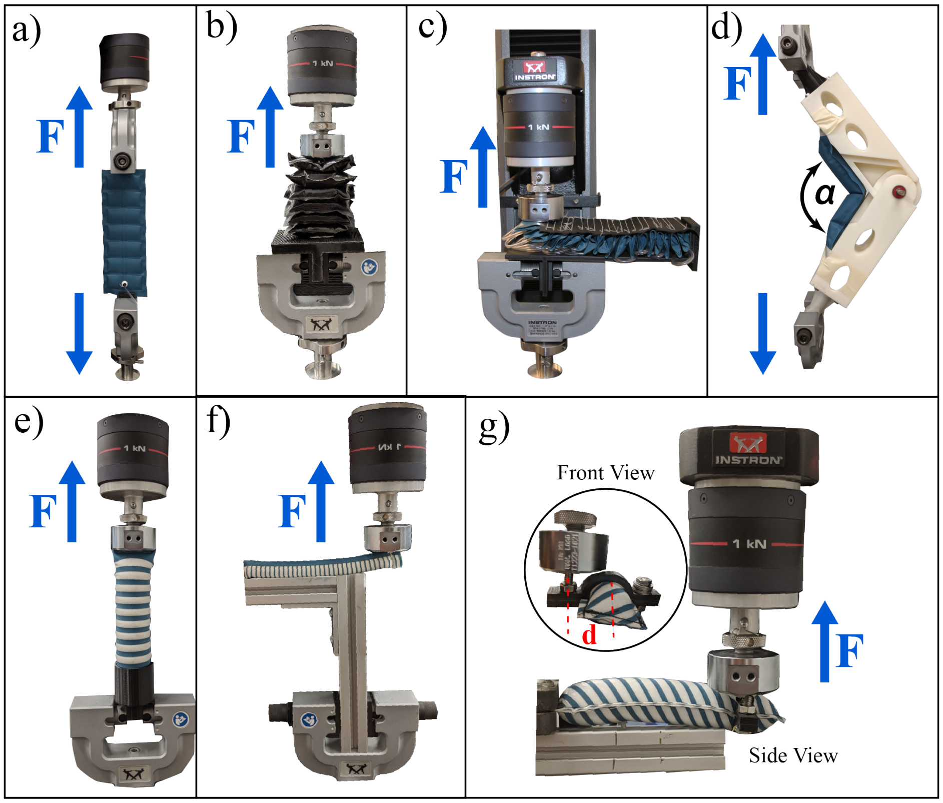

Figure 3. Payload testing experimental setups for a) contracting actuator. b) extending actuator. c) bending actuator. d) stiffening actuator pushing against knee prototype for flexion actuation. e) FRTA extending actuator. f) FRTA bending actuator. g) FRTA twisting actuator.

Frequency Test

To compare how rapidly the three actuators bend, we actuated each actuator with a regulator (ITV1050-21NCl4, SMC, CA), which was set to control the maximum inflation pressure to 0.207MPa, at a maximum flow rate of 200 L/min (Supplementary Video 5).

To determine the maximum frequency of the three actuators, we connected the actuators to a computer-controlled valve (MHE3-MS1H-3/2G-1/8-K, Festo, Esslingen, DE) for equal variations of inflation and deflation (the system would vent to atmosphere) speeds. The tubing connected to each actuator had a smaller inlet diameter of 0.1563mm. In this work, we

Bending Actuator Type	Frequency
Elastomeric Actuator	$2Hz$
Knit FRTAs	$0.7Hz$
Woven FSPAs	$0.45Hz$

Table 3. Frequency Test Summary

maintained the duty cycle to 50% (equal durations for pressurizing and venting to atmosphere) for the frequency tests. The elastomeric actuator's maximum operable frequency was $2Hz$ at the regulated pressure, as seen in Supplementary Table 3 and Fig. 4. The woven FSPA and knit FRTA's maximum operable frequency was $0.5Hz$ and $0.7Hz$ at the regulated pressure, respectively as as seen in Supplementary Table 3 and Fig. 4. Higher frequencies did not allow the actuator to return to its original position.

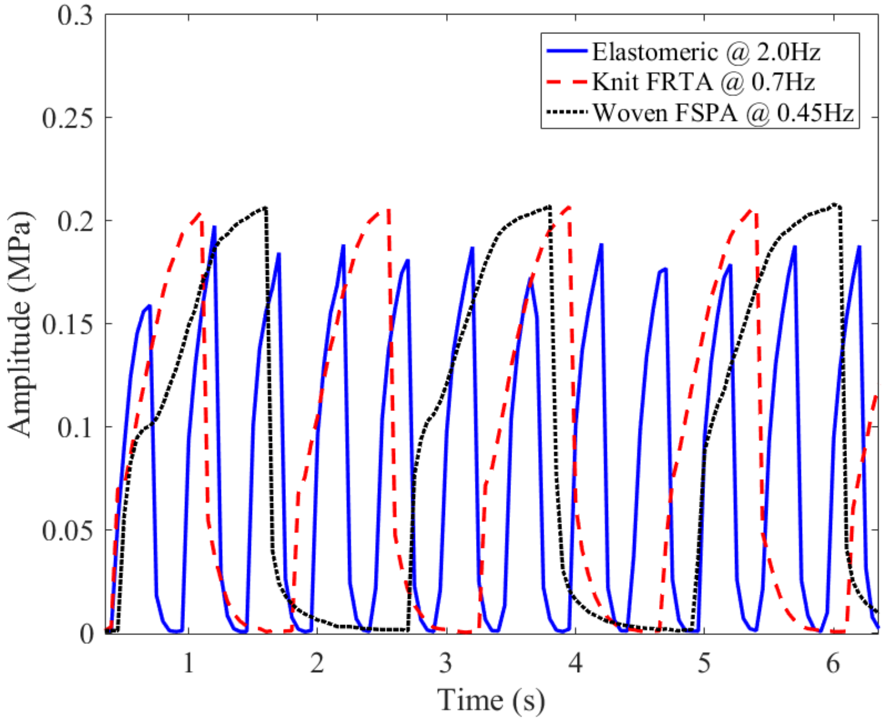

Figure 4. Frequency test for bending actuators.

In Supplementary Video 5, we notice that the woven FSPA actuator has difficulties recovering to its initial position due to the low-stiffness at its strain-limiting layer. We also notice that the elastomeric SPA experiences oscillations when going back and forth between the minimum and maximum pressure due to its material properties. This oscillation however, is not noticed for the knit FRTAs during its inflation and deflation cycles.

In order to improve the overall frequency of the actuators, we can increase the inlet size of the connectors, to improve flow in and out of the actuator. We can also modify the duty cycle, for example by shortening the pressurization period along with a longer venting period.

Efficiency Test

To analyze the external energy interactions of the three bending actuators, we created an experimental setup as seen in Supplementary Figure 5. The energy-in is given by the volume input from the air cylinder, that results in a pressure change (P) recorded by a pressure sensor (ASDXAVX100PGAA5, Honeywell International Inc., Morris Plains, NJ). The volume of air into the system was calculated by the number of strokes multiplied by the volume of the air cylinder per stroke (V). The inertia of the air cylinder plunger is not accounted in this work. The external interaction in the system is measured by the work done

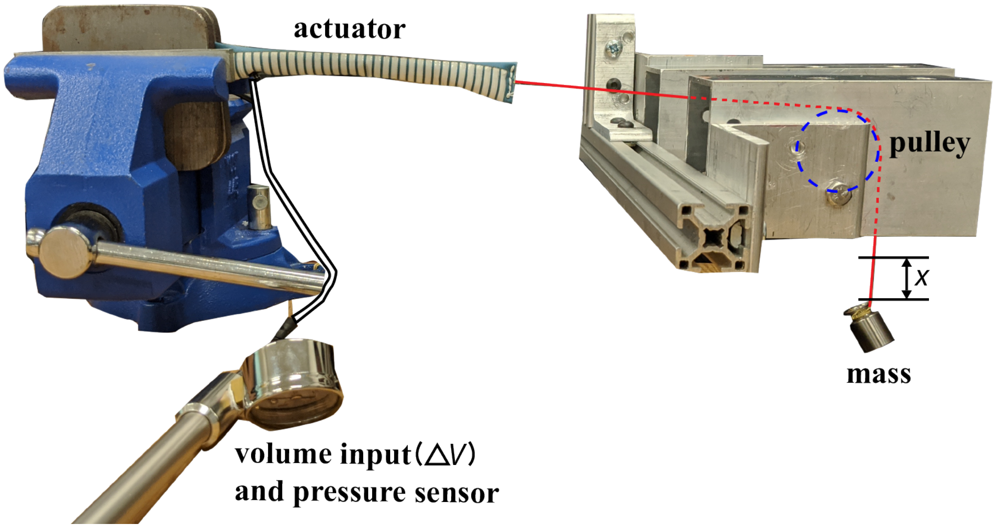

Figure 5. Efficiency test for bending actuators. A total of three trials were run for each mass, per actuator, and averaged.

calculated through the magnitude and displacement of the mass (m) at the end of the string, which is draped over a pulley. We varied the mass (m) with $0.01kg$, $0.02kg$, $0.05kg$, $0.1kg$, $0.2kg$, $0.3kg$, $0.4kg$, and $0.5kg$ weights. A total of three trials were run for each mass, per actuator, and averaged. The rest of the energy is routed to the actuator, or the energy storage. The distance from the actuator and the center of the pulley is equal between the different tests with various actuators. The output work done on the different masses is the product of the mass (m), the acceleration of gravity (g) and the change in the height of the mass (x), as seen below:

$$Efficiency(\%) = \frac{mgx}{P\Delta V} \tag{19}$$

It is important to remember that each actuator was made of different material stiffness and have different wall thicknesses. The elastomeric, woven FSPA, and knit FRTA have a wall thickness of $2mm$, $0.21mm$, and $0.2mm$ respectively.

From the overall efficiency tests, the elastomeric actuator, woven FSPA, and knit FRTA has maximum efficiencies of 0.785% at $0.05kg$, 0.287% at $0.1kg$, and 0.26% at $0.kg$, respectively as seen in Supplementary Table 4. Which means the majority of the energy is engaged in between the mechanical and pneumatic domains. The mechanical domain ideally restores the elastic deformation and dissipates energy through hysteresis. The material properties and wall thickness of the materials play a part here. The pneumatic domain is released to the atmosphere, which means energy can be increased if the pneumatic energy that is stored is vented to an intermediate pressure and cycled back into the input.

We instantly notice that the efficiency of the elastomeric actuator, which has a larger wall thickness, is higher than the fabric actuators when low work done. However, with the increasing work done, the elastomeric actuators' efficiency drops drastically, as seen in Supplementary Figure 6. At the highest work done tested in these experiments, the knit FRTAs, has higher stiffness but lower wall thickness, showed more ideal energy efficiency.

Mass	Elastomeric Actuator Efficiency (%)	Woven FSPAs Efficiency (%)	Knit FRTAs Efficiency (%)
$0.01kg$	0.025± 0.0020945	0.054± 0.0012927	0.031± 0.00054
$0.02kg$	0.378± 0.0103194	0.125± 0.0034538	0.053± 0.002972
$0.05kg$	0.785± 0.0187798	0.268± 0.0057794	0.1± 0.005584
$0.1kg$	0.626± 0.0198147	0.287± 0.0038699	0.216± 0.009608
$0.2kg$	0.451± 0.0070459	0.228± 0.0017158	0.26± 0.006038
$0.3kg$	0.339± 0.0040906	0.202± 0.0037387	0.251± 0.004127
$0.4kg$	0.235± 0.0020313	0.183± 0.0055319	0.214± 0.003827
$0.5kg$	0.193± 0.0018367	0.178± 0.0074668	0.213± 0.004074

Table 4. Efficiency Test for three types of bending soft pneumatic actuators.

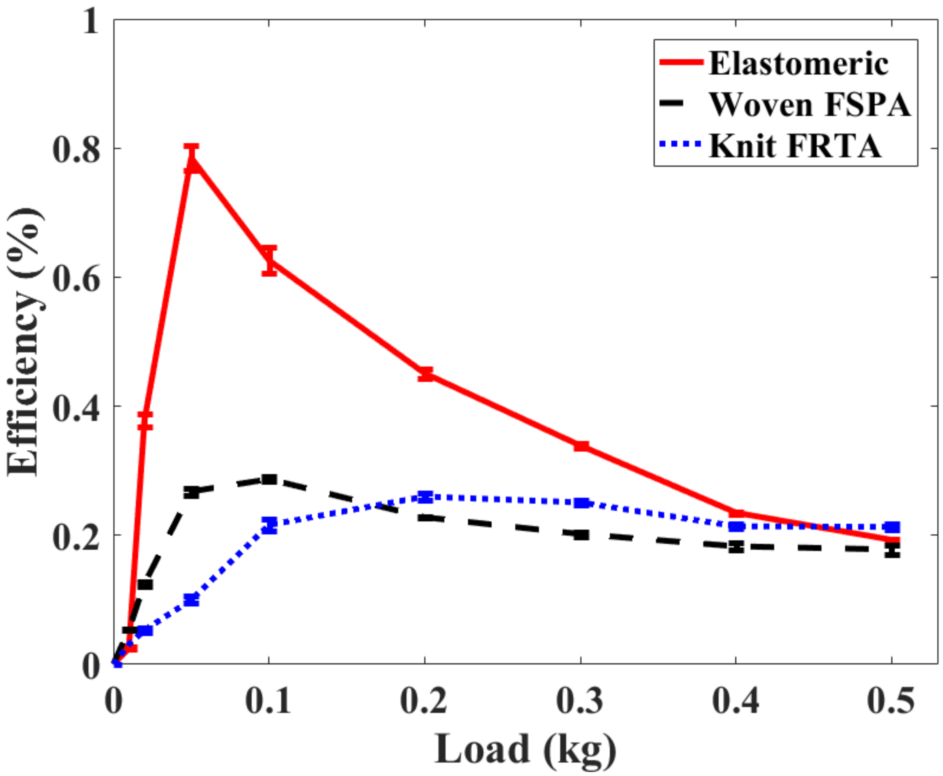

Figure 6. Efficiency test for bending actuators.

From comparison with previous work [27][28], that showed efficiencies for elastomeric actuators to be approximately 1% and 2%, which is relatively similar to what was tested in this work. We have provided insight into the energy efficiency of fabric soft pneumatic actuators. However, we did not account into the initial volume as a factor that could possibly affect the energy routing in our system, this should be further tested especially because our fabric soft pneumatic actuators have close to zero initial chamber volume. We will also look towards dynamic motion and its effect into the internal energy interactions in the future.
